# Overdiagnosis of dental caries in South Korea: a pseudo-patient study

**DOI:** 10.1186/s12903-024-05061-4

**Published:** 2024-12-04

**Authors:** Ji-Young Son, Yuyi Park, Ji-Yeon Park, Min-Ji Kim, Dong-Hun Han

**Affiliations:** 1 Department of Preventive and Social Dentistry, School of Dentistry, Seoul National University, Seoul, Korea; 2 Dental Research Institute, Seoul National University, Seoul, Korea; 3 Department of Dental Education, School of Dentistry, Seoul National University, Seoul, Korea; 4 Department of Dental Hygiene, Dongseo University, Busan, Korea

**Keywords:** Dental caries, Dentist, Overdiagnosis, Pseudo patient, Korea

## Abstract

**Background:**

To evaluates the tendency of South Korean dentists to over-diagnose clinically healthy teeth in pseudo-patients.

**Methods:**

We conducted a pseudo-patient, cross-sectional study in 196 private dental clinics with 58 pseudo-patients in South Korea between August and December 2018. Trained pseudo-patients with no previous oral diseases, including dental caries, diagnosed by two experienced dentists, were sent to each dental clinic. Before visiting each private dental clinic, participants were instructed to state, “I have no symptoms, but I would like to have a dental caries examination”. The oral examination was performed using visual and tactile inspection methods only. The interactions between the dental clinic staff and the pseudo-patient were documented on a data collection form shortly after each visit.

**Results:**

In 33.2% (65/196) of these interactions, the pseudo-patients were diagnosed as having no dental caries. 11.7% (23/196), 12.8% (25/196), 10.7% (21/196), and 10.7% (21/196) of the sample were diagnosed with dental caries in one, two, three, and four teeth, respectively. Dentists diagnosed five or more dental caries in 20.9% (41/196) of the sample. 196 dental clinics diagnosed a total of 503 dental caries. Of these, 392 were in molars. Small solo practice dentists diagnosed 3.54 dental caries and large group practice dentists 1.57, but the difference was not significant (*p* = 0.07). The recommendation rate for dental caries treatment was highest among 43 (55.1%) large solo practices, and lowest in 7 (33.3%) large group practices. However, small solo practices had the lowest rate of preventive care recommendations at 12 (30.8%) and 10 (47.6%) in large group practices. The data shows that preventive care recommendations increased as the practice size increased.

**Conclusion:**

The study findings indicate that Korean dentists tend to over-diagnose dental caries, which could pose a threat to public health both in Korea and worldwide. Therefore, it is important to carefully consider strategies to improve the correct diagnosis and standard of care for dental caries by private dentists.

## Background

In contemporary medical practice, early detection of symptoms before the onset of a disease is considered critical in healthcare management [1–4]. Even for individuals with symptoms, regular health check-ups contribute significantly to overall health promotion [2]. Extensive research on overdiagnosis has been actively pursued in adult healthcare, and recently, scientific methods have emerged to redefine this term and concept [5]. In dentistry, patients with dental caries may require treatment even if they do not exhibit symptoms, due to the presence of clinically visible non-cavitated lesions. Early detection should assess the activity of these lesions to determine the necessity of treatment [6]. Failure to differentiate between active and inactive lesions can lead to overdiagnosis, which may result in overtreatment [7]. An ideal treatment involves prevention and minimal intervention, with early detection of non-cavitated lesions significantly reducing patient time and costs compared to treating cavitated lesions [8].

Dental caries is the most common oral health condition, affecting people of all ages and impacting their quality of life [9, 10]. Untreated dental caries in primary teeth during childhood affects approximately 500 million children, making it the most prevalent chronic condition in this age group [11]. Several studies have proposed the importance of early detection for various oral health conditions [12, 13], including dental caries [14–18]. Dental caries is a non-infectious disease caused by biofilm on the surface of teeth. This biofilm undergoes continuous cycles of mineralization and recalcification [19]. Dental caries initially presents as mineral loss over time, beginning with small enamel lesions and advancing to severe caries that may extend into the dentin [16, 20, 21]. If the dental caries lesion is determined to be active, treatment is necessary [22]. However, if the lesion is deemed inactive and arrested, no treatment is required [22]. Lesions that exist in an intermediate stage, transitioning between active and inactive states, present a challenge in accurately discerning their status [23]. However, the use of visual classification systems enables the adequate detection of early-stage dental caries [24]. ICDAS (International Caries Detection and Assessment System) criteria has demonstrated significant reproducibility and accuracy in evaluating primary coronal caries lesions [25], allowing adequately trained dental professionals to visually detect early lesions [26, 27]. If lesions are treated unnecessarily, it can result in overtreatment. Therefore, a clear diagnosis based on the activity of the lesions according to the ICDAS criteria is essential to prevent overtreatment. However, failure to adhere to ICDAS criteria and overreliance on inadequate education, training, and excessive reliance on radiographs can lead to overdiagnosis [14, 28]. Invasive operative surgery procedures involving drilling can cause patient stress and anxiety, often associated with overdiagnosis [29]. Therefore, the removal of carious regions should be approached with caution. Furthermore, excessive diagnostic procedures may result in overdiagnosis, leading to unnecessary treatments and economic losses for individuals and stakeholders [5, 30–32].

The specific approach employed in dental practice and clinical trials focuses on identifying lesions at the cavitation stage and offering interventions primarily for restorative procedures [33]. Traditional invasive operative surgery primarily based on a ‘drill and fill’ model lack comprehensive preventive and therapeutic management [34]. Dental overtreatment often results from a lack of understanding of caries progression and diagnostic criteria by dental professionals, leading to an indiscriminate approach to both active and inactive lesions [35]. Treating all identified lesions with invasive procedures further exacerbates the problem [35]. This approach may not provide patients with significant benefits in halting the progression of early caries lesions [36]. Dental overdiagnosis of caries is due to a combination of factors, including overlooking caries lesion activity, practice influenced by commercial interests, and inadequate education on modern caries management approaches such as ICDAS criteria. Overtreatment due to overdiagnosis is particularly prone to invasive surgery and results from a failure to consider the activity of carious lesions and an inability to apply modern approaches to carious lesion management in clinical practice. Instead, it results from a reliance on restorative treatments following cavity preparation. This appears to be due to ethical lapses among for-profit dentists and inadequate continuing education. Therefore, this study aims to determine the incidence of overdiagnosis in South Korea and to investigate the factors associated with overdiagnosis.

## Methods

### Study setting, study design, participants

This study was an experimental study aiming to use Visual-tactile caries lesions diagnosis as an intervention and caries lesions’ diagnostic and treatment decisions as the outcome. The study was carried out from August 2018 to December 2018 in Seoul and Busan which are the largest and second largest metropolis in Korea. The study enrolled registered private dental clinic dentists as participants. The total sample size of 196 dental clinicians in the study was determined by calculating a 7% sample error and 95% confidence interval from those registered in the Seoul and Busan metropolitan areas.

### Ethics considerations

The study protocol was approved by the Dongseo University Bioethics Committee, with approval number 2018-008-HR-02. The study was conducted according to the ethical standards outlined in the Declaration of Helsinki of 1964 and its subsequent revisions. Voluntary written informed consent was obtained from all pseudo-patients.

### Pseudo-patient recruit criteria

We selected pseudo-patients based on the following criteria.

(1) No subjective symptoms of oral diseases such as dental caries or periodontal disease; (2) Adequate cognitive abilities and knowledge to comprehend the diagnosis and treatment plan presented by the dentist; and (3) Individuals aged between 19 and 22 years.

### Pseudo-patient screening

Sixty-three pseudo-patients were recruited based on the previously mentioned criteria from May 2018 to August 2018. Convenience and snowball sampling methods were utilized to recruit the pseudo-patients. Each pseudo-patients underwent two oral examinations by two dentists with at least ten years of experience. One dentist has participated and calibrated for 13 years in the Korean National Health and Nutrition Examination Survey and the National Oral Health Survey conducted by the Ministry of Health and Welfare. The other dentist has practiced for 10 years in a private dental clinic. We have modified the World Health Organization (WHO) and ICDAS criteria to create the following new criteria for determining dental caries. Since the purpose of this study is to determine whether teeth with sound enamel surface are diagnosed as caries, not whether teeth with cavity formation are diagnosed as caries, codes 0–2 were selected according to the full ICDAS criteria and codes 3–5 according to WHO criteria for the efficiency and convenience of the study.

code 0: normal.

code 1: white spot (demineralization).

code 2: enamel caries with dark pigmentation.

code 3: distinct cavity in enamel.

code 4: dentin-involved caries.

code 5: pulp-exposed caries.

Based on the above criteria, two dentists assessed the caries status of pseudo-patients lying in a dental chair under an illuminated light using a dental mirror to examine the permanent teeth. The selection criteria for the pseudo-patients were as follows.

1. No caries of code 2 or higher.
2. No dental fillings.

In the previous study, inactive lesions were assessed based on three criteria: (1) The enamel surface exhibits whitish, brownish, or black discoloration; (2) The enamel may display shine and feels hard and smooth upon probing; (3) On smooth surfaces, caries lesions are typically positioned at a distance from the gingival margin [23]. Our examination categorized as follows: when there was no obvious plaque and no white lesion on air-drying, it was classified as 0; if a white lesion was observed upon air-drying, it was classified as 1. Cases with evident cavity or the presence of plaque were excluded from the study. The final 58 pseudo-patients were selected by duplicate oral examinations arranged with two dentists. After being examined by two dentists, pseudo-patients visited the dental clinic within a week. They were reminded via cell phone text to brush their teeth before visiting the dental clinic.

### Pseudo-patient scenario

The scenarios were developed by the researchers of this study. The pseudo-patients were assumed to have no oral symptoms, such as toothache, but to visit the dentist for a routine oral examination. For the same oral examination setting, intraoral and/or panoramic radiographs were not allowed. We created a scenario in which the pseudo-patients would not accept treatment, even if the pseudo-patients offered the treatment immediately after an oral examination.

The pseudo-patients were trained as follows.

1. When they visit a dental clinic and say, “I do not have any toothache, but I would like to have my teeth checked for dental caries.”
2. If the dentist recommends an X-ray for additional information, say, “I would like to have an oral examination without X-rays.”
3. If, after an oral examination, the dentist finds that you have a dental caries and recommends that you have it treated today, say “I will not have it treated today.”

The words of each pseudo-patient during the interaction with the dentist are designed for the following reasons.

1. To exclude the diagnosis of caries based on symptoms.
2. To limit the caries diagnostic tools to visual and tactile sensation and to avoid harm to the pseudo-patient from possible radiation exposure.
3. To avoid potential harm to the pseudo-patient due to overdiagnosis and subsequent overtreatment.

### Data Collection

Seoul and Busan were divided into five regions (East, West, South, North, and Center). A stratified convenience sampling approach was employed to divide Seoul and Busan into five regions, which were then categorized into three distinct zones: central business district, regional business district, and residential district. Each of the divided areas was designated, and pseudo- patients select and visit three to four dental clinics that were most convenient for them. No hospitals or dentists were selected as duplicates. Table 1 presents the number of dental clinics visited and pseudo-patients for each city. Immediately following the oral examination at the visited dental clinic, oral examination results were recorded electronically as oral diseases (number and location of dental caries, periodontal disease), treatment plans (cavity filling, endodontic treatment, periodontal treatment, topical fluoride application, minor oral surgery, impacted third molar extraction), and the size of the visited dental clinic by the number of dentists. Additionally, following the study, focus group interviews were conducted with five pseudo-patients in Seoul, representing 10% of the total sample, to explore their perspectives on overdiagnosis.

**Table 1:** General characteristics of study participants

	Pseudo-patients allocated	Dental clinics visited	Number of dental clinics in 2018 [KIm and Kim, 2018]
	*n*(%)	*n*(%)	*n*(%)
Seoul	41 (70.7%)	121 (61.7%)	4,807 (79.2%)
Busan	17 (29.3%)	75 (38.3%)	1,265 (20.8%)
Total	58 (100.0%)	196 (100.0%)	6,072 (100.0%)

### Study variables

The largest group of dental clinics consisted of solo practices, followed by dental clinics with two dentists as shown in Table 2. Dental clinics were categorized by their workforce size according to the Economic General Survey of Korea [37]. The size classification consisted of four categories: small solo practice (1 dentist and under 5 dental chairs), large solo practice (1 dentist and 5 or more dental chairs), medium group practice (2 to 4 dentists), and large group practice (5 or more dentists). There were 39 small solo practices (19.9%), 78 large solo practices (39.8%), 58 medium group practices (29.6%), and 21 large group practices (10.7%).

**Table 2:**
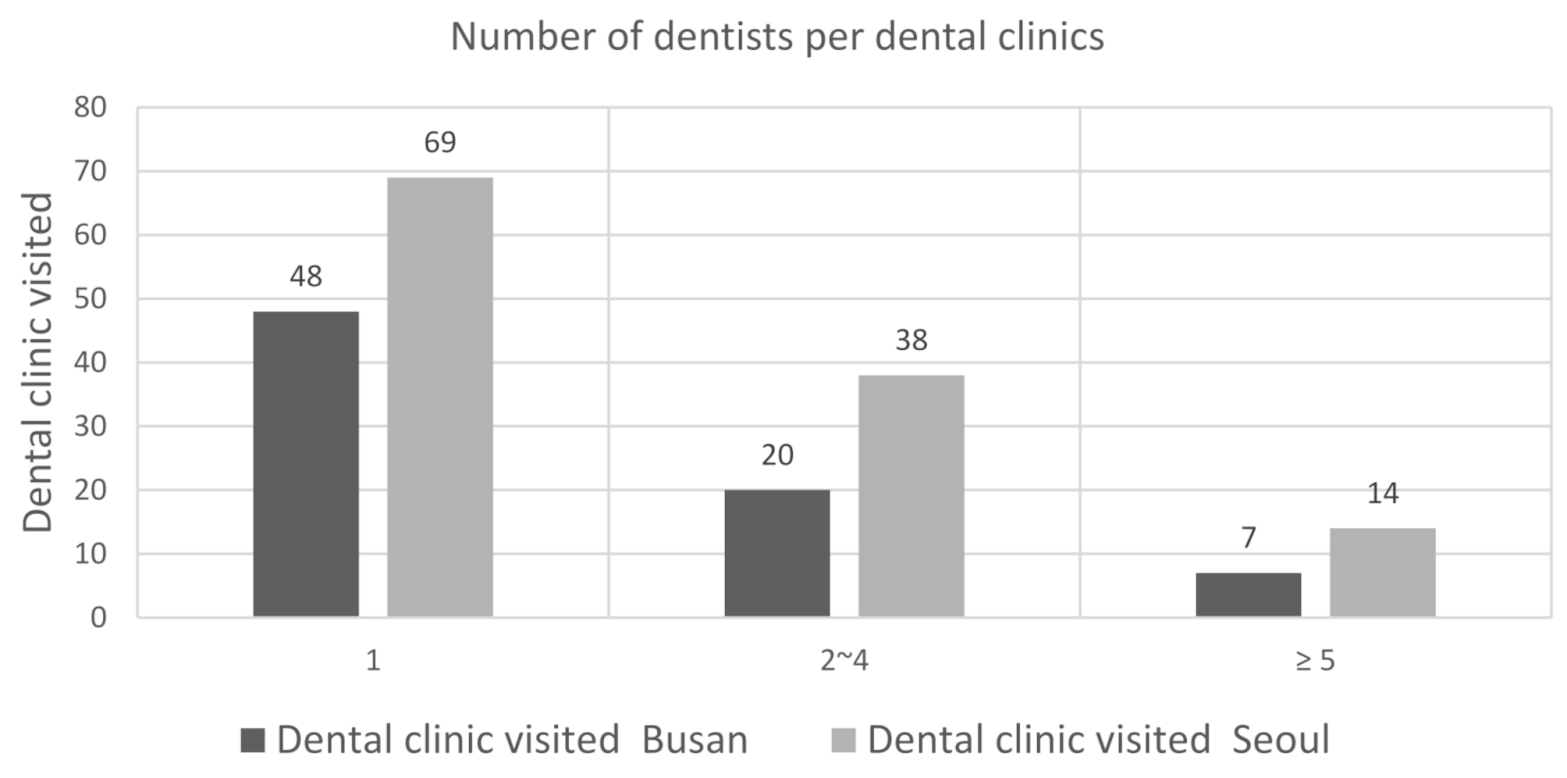
Size of dental clinics

We categorized the number and location of dental caries diagnosed by each dentist. In addition to the dentist’s diagnosis of dental caries, we assessed whether the patient was advised to receive dental treatments such as cavity filling, endodontic treatment, periodontal treatment, minor oral surgery, and impacted third molar extraction. We also looked at whether there was a recommendation for preventive treatments for dental caries, such as fluoride treatments or sealant.

### Statistical analyses

The general characteristics of the dental clinics visited and pseudo-patients, and the number of dental caries diagnosed were presented by frequency analysis. Kruskal–Wallis nonparametric test was applied to analyze the relationship between dental caries and the size of dental clinics. The treatment plans were analyzed by chi-square tests based on the size of the clinics. Significance level was set at 95%. Data was analyzed using SPSS 24.

## Results

Table 1 shows general characteristics of study participants. A total of 196 dental clinics were visited by the pseudo-patients. Among them, 121 clinics (62.7%) were in Seoul and 75 clinics (38.3%) in Busan. According to the statistics of the National Health Insurance Service [38], there were 4807 dental clinics (79.2%) in Seoul and 1265 (20.8%) in Busan in the fourth quarter of 2018. Recruiting dentists solely based on the distribution of dental clinics in Seoul and Busan in Korea in 2018 would have resulted in underrepresenting dentists in Busan. Therefore, this study recruited a higher number of dentists in Busan.

Of the 196 dental clinics that were visited, 117 (59.7%) were operated by a single dentist, with 69 in Seoul and 48 in Busan. There were 48 (24.5%) dental clinics that had two or three dentists, with 38 in Seoul and 20 in Busan. In addition, 31 (15.8%) dental clinics were staffed with four or more dentists working as a group, with 14 in Seoul and 7 in Busan (Table 2).

Of the 196 dental clinics, 65 (33.2%) agreed with the researchers that there was no dental caries. 24.5% of dental clinics diagnosed one or two cases of non-cavitated or cavitated dental caries requiring invasive operative treatment, followed by 21.4% for three to four cases, 13.8% for five to six cases, 3.6% 191 each for seven to eight cases and nine 192 or more cases (Table 3).

**Table 3:** The number of dental clinics according to the number of dental caries diagnosis

Number of dental caries diagnosis	Number of dental clinics
*n*	*n*(%)
0	65 (33.2%)
1	23 (11.7%)
2	25 (12.8%)
3	21 (10.7%)
4	21 (10.7%)
5	18 (9.2%)
6	9 (4.6%)
7	6 (3.1%)
≥ 8	8 (4.0%)

The maxillary second molars were most frequently diagnosed with caries (right 32.5%, left 33.2%), followed by the mandibular molars (right 20.8%, left 25.6%) and the maxillary first molars (right 18.7%, left 19.4%). Not a single dentist has ever determined that mandibular anterior teeth have dental caries (Table 4).

**Table 4:** Distribution of dental caries diagnosed by 196 dental clinics

Right								Left							
Mx. 3rd molar	Mx. 2nd molar	Mx. 1st molar	Mx. 2nd premolar	Mx. 1st premolar	Mx. Canine	Mx. Lateral Incisor	Mx. Central Incisor	Mx. Central Incisor	Mx. Lateral Incisor	Mx. Canine	Mx. 1st premolar	Mx. 2nd premolar	Mx. 1st molar	Mx. 2nd molar	Mx. 3rd molar
0/196 (0.0%)	64/196 (32.5%)	37/196 (18.7%)	5/196 (2.8%)	9/196 4.8%)	7/196 (3.5%)	0/196 (0.0%)	33/196 (16.6%)	0/196 (0.0%)	9/196 (4.8%)	0/196 (0.0%)	11/196 (5.5%)	8/196 (4.2%)	38/196 (19.4%)	65/196 (33.2%)	4/196 (2.1%)
0/196 (0.0%)	41/196 (20.8%)	41/196 (20.8%)	14/196 (6.9%)	0/196 (0.0%)	0/196 (0.0%)	0/196 (0.0%)	0/196 (0.0%)	0/196 (0.0%)	0/196 (0.0%)	0/196 (0.0%)	0/196 (0.0%)	15/196 (7.6%)	50/196 (25.6%)	50/196 (25.6%)	3/196 (1.4%)
Mn. 3rd molar	Mn. 2nd molar	Mn. 1st molar	Mn. 2nd premolar	Mn. 1st premolar	Mn. Canine	Mn. Lateral Incisor	Mn. Central Incisor	Mn. Central Incisor	Mn. Lateral Incisor	Mn. Canine	Mn. 1st premolar	Mn. 2nd premolar	Mn. 1st molar	Mn. 2nd molar	Mn. 3rd molar

The average number of diagnoses for dental caries were highest in small solo practices and tended to decrease with practice size; however, this association was not statistically significant (Table 5).

**Table 5:** Mean and standard deviation of dental caries diagnosis by size of dental clinic

Size of dental clinic	Small solo practice *n* = 39	Large solo practice *n* = 78	Medium group practice *n* = 58	Large group practice *n* = 21	*P*
Mean	3.54	2.62	2.59	1.57	0.13

*P value* obtained by Kruskal–Wallis H-test

The large solo, medium group, small solo practitioners were more likely to recommend invasive operative treatment, while large group practitioners were less likely to recommend invasive operative treatment. On the other hand, preventive treatment such as topical fluoride application tended to be most common in large group practitioners, followed by medium group practitioners, large solo practitioners, and then small solo practitioners. However, this association was not statistically significant (Table 6).

**Table 6:** Difference in recommended treatment based on the size of dental clinic

Variables		Recommended treatment									
		Invasive operative treatment of dental Caries *n*(%)			χ^2^	*P*	Preventive treatment *n*(%)			χ^2^	*P*
		No	Yes	Total			No	Yes	Total		
Size of dental clinic	Small solo practice	19(48.7)	20(51.3)	39(100.0)	3.275	0.351	27(69.2)	12(30.8)	39(100.0)	1.754	0.625
	Large solo practice	35(44.9)	43(55.1)	78(100.0)			48(61.5)	30(38.5)	78(100.0)		
	Medium group practice	27(46.6)	31(53.4)	58(100.0)			35(60.3)	23(39.7)	58(100.0)		
	Large group practice	14(66.7)	7(33.3)	21(100.0)			11(52.4)	10(47.6)	21(100.0)		

*P* value obtained by chi-square test

Following the study, focus group interviews were conducted with five pseudo-patients in Seoul, representing 10% of the total sample, to explore their perspectives on overdiagnosis. Two participants reported discomfort and skepticism toward dentists who diagnosed dental caries without evidence, while the remaining three participants viewed the dentists as meticulous and reliable.

## Discussion

Our main finding is that the diagnosis and treatment decisions for caries are extensive. The primary findings of the study suggest that a very large number of dental practices diagnose caries in pseudo-patients, with the highest incidence in maxillary second molars and the lowest incidence in mandibular front teeth. Furthermore, the size of dental clinic had an impact on the average number of dental caries diagnoses per pseudo-patient and treatment planning. Specifically, an increase in the number of dentists was associated with a decrease in caries diagnoses but an increase in preventive care.

The pseudo-patient study is a research study used to evaluate healthcare service quality. The study was inspired by skepticism about psychiatric diagnosis objectivity in the 1970s [39, 40]. It aimed to investigate the ability of mental health professionals to accurately distinguish between individuals with and without genuine mental illness. In the pseudo-patient studies, researchers enlist individuals who feign illness and visit healthcare providers with a designated set of symptoms. The researchers subsequently appraise the quality of care given by the healthcare providers according to a predetermined set of criteria. In order to facilitate comprehension, we present the design of a previously conducted methodological procedure of a pseudo-patient study [41].

**Table:** 

Step	Description
**Study Design**	Pseudo-patients visited 242 pharmacies in Sri Lanka.
**Sample Size**	242 visits conducted; 32 pseudo-patients involved.
**Pseudo-patient Role**	Pharmacy graduates or students presenting symptoms.
**Visit Procedure**	Presented one of 4 infection scenarios (sore throat, cold, etc.).
**Data Collection**	Audio recorded interactions; data sheets completed after visits.
**Request Levels**	3 levels: symptom relief, stronger medicine, direct antibiotic request.
**Pharmacist Check**	Verified by observing license or asking for the pharmacist.
**Expected Outcome**	No antibiotics for viral cases; referral for UTI cases.

There are arguments that the pseudo-patient study is unethical because it involves deceiving healthcare providers [42]. The dentists in the study were unaware of the true identities of the participants and treated them as if they were real patients, raising ethical concerns about informed consent and potential harm to the well-being of the patients. Opponents of the pseudo-patient study argue that it is fundamentally flawed because of its exploitative nature, use of deception, and potential harm to the subjects. It addresses the delicate issue of trust in the professional-patient relationship, which is critical to its effectiveness but also vulnerable to skepticism [43]. The perception of deceiving dentists and not obtaining their consent would have made it very difficult to pass the IRB for this study. The IRB center of the dental school and the IRB center of the dental hospital refused to approve the IRB of the study, so the study was approved by the IRB of a university without a dental school. However, for this study, only non-afflicted patients who received caries-free diagnoses from two dentists in duplicate examinations were selected to visit the dentist. They were instructed to truthfully report the absence of any pain or other symptoms during their interactions with the dentist, so there was no deception.

A pseudo-patient study has several desirable implications for caries diagnosis. Initially, it can evaluate the diagnostic precision of dental professionals in detecting and diagnosing dental caries. This can assist in pinpointing possible flaws or variations in diagnostic methods, resulting in enhanced clinical judgment. Furthermore, by emulating patient scenarios, a pseudo-patient study can expose instances of excessive diagnosis or overtreatment of dental caries. This can increase awareness among dental professionals and encourage evidence-based treatment approaches that prioritize patients. Additionally, data from a simulated patient study can guide quality improvement initiatives, enabling dental practices to strengthen their diagnostic procedures and treatment planning processes. This can ultimately result in improved patient outcomes and more effective resource allocation.

Recent studies utilizing pseudo-patients have investigated the reactions of pharmacists to minor ailments [41, 44]. However, to this point, no pseudo-patient analysis has focused on overdiagnosis aside from two meta-analyses of overtreatment within dental caries [35, 45].

In the field of dentistry, a study was conducted among Boston dentists in 1979 In order to expose the over-use of x-rays by Boston dentists, researchers posing as patients seeking information conducted a telephone survey among dentists. The results showed that dentists interviewed claimed substantially greater use of x-rays than they admitted to in non-deceptive inquiries. This finding was reported in the American Journal of Public Health [46].

Surprisingly, dentists in the major South Korean cities of Seoul and Busan often diagnose dental caries without distinguishing between inactive and active lesions. A few dentists refrained from overdiagnosing or recommending overtreatment. Overdiagnosis of dental caries can have serious implications for both patients and the healthcare system. Firstly, it can lead to unnecessary and invasive dental treatments, including fillings, endodontic treatments, or extractions, which can cause physical and economic burden to patients. These procedures also entail risks and potential complications. Secondly, overdiagnosis of dental caries adds to the overuse of healthcare resources. Dental clinics may allocate their time, staff, and resources to treat conditions that do not require intervention, while neglecting patients with genuine oral health issues. This inefficiency can burden the healthcare system and lead to increased costs for both patients and healthcare providers alike. Lastly, the psychological impact of overdiagnosis should not be overlooked. False positive diagnoses can cause anxiety and stress for patients who may worry about their oral health and the potential consequences of untreated dental caries. After the study, focus group interviews with pseudo-patients revealed mixed reactions to overdiagnosis. Some felt discomfort and skepticism toward the dentists who diagnosed dental caries without evidence, while others viewed the dentists as meticulous and reliable. Therefore, overdiagnosis and overtreatment are not solely issues of the dentists, but rather part of a medical-dental culture co-created by both dentists and patients.

In a study assessing overdiagnosis and overtreat tendencies in clinical situations among final-year Spanish dental students, the majority correctly selected diagnostic criteria, but 41.7% reported unnecessary restorative treatment as their treatment plan in various clinical scenarios [28]. To address this issue, guidelines for the management of dental caries have been developed by experts with an emphasis on prevention and early intervention.

At present, several dental caries classification and management standards are widely used worldwide. The International Caries Detection and Assessment System (ICDAS) was founded in 2002 [47], , and in 2009, dental caries activity tests were included for the development of the modified clinical caries classification standard—ICDAS‑II [48]. The International Caries Classification and Management System, ICCMS, was proposed based on the ICDAS [14]. In USA, the U.S. National Institute for Dental and Craniofacial Research (NIDCR) Early Childhood Caries Collaborative Centers Criteria was developed [49].

Why do dentists overdiagnoses dental caries? Firstly, it may be because in a fee-for-service system, dental practitioners are reimbursed based on the number of services they provide to patients [50]. This payment structure has the potential to create an incentive for overdiagnosis and overtreatment [51]. Under the fee-for-service model, dentists may feel pressure to increase their revenue by diagnosing and treating more dental caries, even when the condition might not require immediate intervention. This could result in a greater chance of overdiagnosis since dentists may be overly cautious to ensure compensation for each identified case of caries. Additionally, fee-for-service reimbursement may prioritize quantity of care over quality, leading dentists to focus on procedures rather than engaging in thorough diagnostic evaluation and shared decision-making with patients [52]. This may lead to excessive treatments, such as fillings or other invasive procedures, that may not be clinically required [52]. Secondly, dentists may have concerns about potential legal consequences if they fail to diagnose and treat dental caries [53]. This concern for liability can prompt them to take a more cautious approach, increasing the likelihood of overdiagnosis. Thirdly, due to inadequate training in proper ICDAS criteria either through formal education course or lifelong learning, dentists tend to rely excessively on radiographs for caries diagnosis rather than adhering to the ICDAS criteria [14, 28]. Finally, patients often seek dental care with the expectation of a comprehensive oral health evaluation and treatment of any issues [54]. Dentists may sense pressure to fulfill these expectations, which can result in overdiagnosis if even minor indications of caries are perceived as warranting treatment.

Although the association was not statistically significant in our study, dental clinics with more dentists tended to diagnose less caries and suggest more preventive treatments. More structured simulated pseudo-patient studies are needed to test the hypothesis that having more peers to work with may lead to a surveillance-conscious refrain from overdiagnosis and overtreatment. In addition, of the results that were not statistically significant in this study, no previous research has been conducted on the topic of whether commercial interests may increase caries diagnoses in private practices with one dentist but multiple chairs, and further research is needed.

This study has several limitations. First, we sampled dental clinics in only a few regions of South Korea to confirm our findings. Second, we sampled dental clinics in Seoul and Busan, two of the largest cities in South Korea, by region, using a convenience sample could introduce bias. In addition, the future studies would be enhanced by the inclusion of a larger sample size and a longer time period. Also, the sampling of dentists and dental clinics was not conducted in duplicate, thus it is unclear whether the dentists examined dental caries consistently. Third, although the control patients recorded their data immediately following the checkup, they relied on their memories which may be subject to recall bias. Fourth, we were unable to conduct probing on the pseudo-patient for swift and efficient oral examination. The assessment relied solely on the dentist’s visual inspection and the presence of white spots observed during air-drying. However, the presence of plaque or evident cavitation led to their exclusion. Fifth, patients were selected based on the absence of current caries. Risk factors for caries lesions, such as dietary habits or brushing frequency, were not assessed. Considering the patients’ caries risk would have enabled a more precise determination of the factors contributing to diagnosis and treatment. Additional research is necessary in this field. Finally, although caries diagnoses were recorded on a tooth-by-tooth basis, only the necessity of treatment was documented. Therefore, detailed analysis of caries not requiring treatment on a tooth-by-tooth basis was not possible. Despite these limitations, the study shows that Korean dentists tend to overdiagnose caries, indicating a problem.

Addressing the issue of overdiagnosis of caries requires ethical reflection within the dental profession [55]. This involves promoting patient-centered care, ensuring informed consent, advocating for evidence-based practices, and developing guidelines that prioritize clinical necessity over financial incentives [55]. It is crucial to strike a balance between preventive measures and unnecessary interventions to uphold the ethical principles of beneficence, non-maleficence, and patient autonomy in dental practice [56].

It is important for dental healthcare systems and policymakers to consider alternative payment models that prioritize quality, value, and patient outcomes [57]. This will minimize the overdiagnosis of dental caries and ensure that patients receive the most appropriate and necessary care.

Healthcare providers must offer patients impartial information regarding their treatment options [58]. Being fully informed about the pros, cons, benefits, and risks of the care they will receive is an important first step in ensuring that they feel empowered [58].

While there is no doubt that many healthcare services have positive effects, there are also some that are unnecessary or even counterproductive. However, attempts to determine the extent of overdiagnosis and overtreatment are still in their infancy. The risk of overdiagnosis is thought to be high in South Korea because the healthcare system is easily accessible and has inherent incentives to provide more services [59]. A wise approach is needed to ensure that medical technology is used efficiently and effectively where it is needed [60]. Incentivizing correct diagnosis and treatment based on the ICDAS guidelines in actual clinical practice could potentially reduce overdiagnosis and overtreatment in the future. Therefore, early and lifelong training through education on the ICDAS guidelines for the diagnosis and treatment of dental caries is imperative. However, traditional dental caries detection methods, including visual and tactile sensations based on ICDAS guidelines, are subjective and may vary depending on the practitioner [61, 62]. Therefore, incorporating caries detection dyes into a standardized protocol could enable more accurate diagnosis. Staining demineralized dentin can be useful for identifying areas that are difficult to detect visually or tactilely, such as prepared undercuts or the dentino-enamel(DE) junction [62, 63].

This study highlights the significant prevalence of dental caries overdiagnosis in South Korea, underscoring the need for action. To address this, it is crucial to communicate the findings to local dental associations and health policy committees to strengthen diagnostic criteria and improve practice guidelines. Enhancing university curricula with continuing education for future dentists and promoting awareness campaigns for practitioners and patients will further help prevent overdiagnosis and overtreatment by ensuring adherence to standardized protocols and clinical guidelines.

## Data Availability

The data supporting the study findings are not publicly available to protect confidentiality of the research participants. Upon reasonable request, the data can be made available. For any additional inquiries, please contact the corresponding author.

## Abbreviations

ICDAS: International Caries Detection and Assessment System
WHO: World Health Organization
